# Magnetic and electromagnetic methods in reducing microbial contamination in water: A systematic review and meta-analyses

**DOI:** 10.1016/j.onehlt.2025.101213

**Published:** 2025-09-20

**Authors:** Shiva Shahveh, Fatemeh Razaghi, Maziar Naderi

**Affiliations:** a Department of Microbiology, Faculty of Sciences, Karaj Branch, Islamic Azad University, Karaj, Iran; b Department of Environmental Health Engineering, Faculty of Public Health, Tehran University of Medical Sciences, Tehran, Iran

**Keywords:** Water disinfection, Magnetic methods, Electromagnetic methods, Microbial contamination

## Abstract

Recent advancements in water disinfection methods have introduced magnetic and electromagnetic techniques as potential solutions. However, the efficacy of these methods remains inconsistent and necessitates thorough evaluation. This meta-analysis aimed to evaluate the effectiveness of magnetic and electromagnetic methods in reducing microbial contamination in water by synthesizing findings from relevant studies. A comprehensive literature search was performed across multiple electronic databases, including Web of Science, PubMed, and Scopus, to identify studies published between January 2010 and March 2025. Data extracted from eligible studies were analyzed using meta-analytical techniques to determine overall effectiveness, with heterogeneity assessed through effect sizes. The results revealed mixed efficacy for magnetic and electromagnetic methods in water disinfection. While several studies reported negative effect sizes, including a notable negative effect size of −0.8 (CI: −1.2, −0.4), others demonstrated positive effects, with an effect size of 0.7 (CI: 0.2, 1.2) indicating significant improvements in disinfection efficacy. The findings demonstrated that magnetic and electromagnetic methods can be valuable tools for microbial control in water treatment. Moreover, magnetic and pulsed fields indicated potential for environmental remediation and enhancing antimicrobial efficacy. However, the observed increase in microbial populations following exposure to magnetic fields proposes the necessity for optimized conditions to prevent unintended microbial proliferation. This study emphasized the importance of integrating innovative disinfection methods into comprehensive water management strategies that aligns with One Health principles, ultimately supporting healthier ecosystems and communities.

## Highlights

• Magnetic and electromagnetic methods can effectively disinfect water and control microbes.
• Magnetic fields can influence microbial growth, promoting or inhibiting it based on conditions.
• Pulsed fields demonstrate promise in environmental remediation and enhancing antimicrobial efficacy.
• Optimizing conditions is crucial to prevent unintended microbial proliferation post-exposure.
• Variability in outcomes suggests need for further research and methodological standardization.

## Graphical abstract

**Figure:**
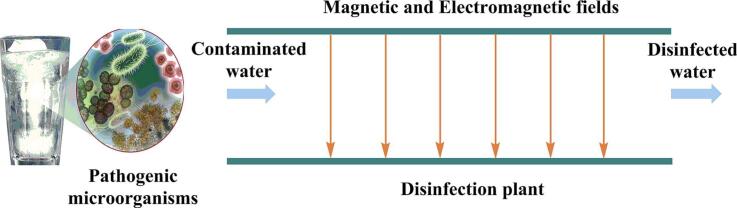

## 1 Introduction

Water is an essential resource that maintains human health, supports animal habitats, and maintains the integrity of the environment. The interrelationship of human, animal, and environmental health is embedded in the One Health framework, emphasizing that the health of all three domains is inherently interconnected [1]. The microbial contamination of water supplies poses significant risks in these domains, leading to waterborne diseases in humans, infections in animals, and disruption of ecosystems. Ensuring access to safe and clean water is therefore a critical component of the One Health approach, which has broad implications for public health, animal welfare, and environmental sustainability [2].

Traditional water disinfection methods, such as chlorination and chemical treatment, while effective, often have disadvantages, including the formation of harmful by-products and environmental pollution [3]. As a result, there is growing interest in innovative and environmentally friendly disinfection technologies that can mitigate these issues. Among these, magnetic and electromagnetic field-based methods have emerged as promising alternatives. These technologies can protect human populations from waterborne diseases, ensure animal health, and preserve aquatic ecosystems [[4], [5], [6]].

The technologies for water disinfection involve the use of magnetic fields to influence the behavior of microorganisms in water, resulting in their inactivation or elimination [7,8]. These methods can include the use of magnetically activated nanomaterials, magnetohydrodynamics, and magnetic resonance technologies [5]. The basic principle is that magnetic fields can modify the physical and chemical properties of water, enhancing the efficiency of existing disinfection processes, or activating new mechanisms of microbial inactivation [6,9].

Advantages of magnetic field-based technologies include their potential for energy efficiency, reduced chemical usage, and minimal production of harmful disinfection byproducts [10,11]. Furthermore, these methods can be integrated into existing water treatment infrastructure, providing a versatile solution to water disinfection challenges. However, challenges exist, including the need for extensive research to fully understand the mechanisms involved, variability in efficacy depending on water quality, and the potential for high initial investment costs [5,12].

The mechanisms by which magnetic and electromagnetic fields exert their disinfection effects are multifaceted and have not yet been fully characterized [13]. Proposed mechanisms include disruption of microbial cell membranes, production of reactive oxygen species (ROS), and enhancement of coagulation processes [14]. Understanding these mechanisms is crucial for optimizing the application of magnetic fields in water disinfection and addressing the diversity of microbial resistance [4,15].

Several countries have begun exploring the application of magnetic fields in water treatment. For example, pilot projects in areas facing severe water quality challenges have shown promising results [16]. Reports from countries such as India and Brazil indicate that while magnetic field technologies can significantly reduce microbial loads, further studies are needed to assess their long-term efficacy and economic viability [17,18].

Various studies have investigated the efficacy of magnetic field-based technologies for water disinfection. Molouk et al. evaluated the effect of magnetic fields on the physical, chemical and microbiological properties of the lake water in Saudi Arabia. The results of this study showed that magnetic fields play a major role in solving many environmental problems such as microbiological contaminations [19]. Similarly, Pina et al. carried out a study on the exploring the potential of magnetic antimicrobial agents for water disinfection. The results of their study showed that the magnetic antimicrobial device was able to successfully disinfect water [20].

In another study, Varkey et al. conducted a study on the disinfection of *E. coli* in water using moderate electric and magnetic fields. The results of their study showed that moderate alternating current (AC) electric fields (10 V/cm to 1 kV/cm) and moderate static magnetic fields (10 mT to 65 mT) could significantly kill *E. coli* by 90 % or more [21]. Moreover, Liu et al. investigated the sterilization effect of magnetic fields on heterotrophic bacteria in circulating cooling water. Their results demonstrated that the bacterial inhibiting effect was 7.22 % ∼ 20.35 % higher when the direction of flow of water was in parallel with the magnetic fields than in anti-parallel [22].

These studies have reported varying degrees of success, with some showing significant reductions in microbial counts, while others have raised concerns about the reproducibility of results and the need for standardized protocols [23]. The diversity in methodology and experimental conditions highlights the complexity of evaluating the effectiveness of these technologies [24].

Despite the growing body of literature, significant gaps remain in understanding the underlying mechanisms, optimizing operational parameters, and assessing the long-term effects of magnetic field-based disinfection [25]. Furthermore, the heterogeneity of results across studies underscores the need for systematic reviews to synthesize findings and identify best practices [26].

Conventional disinfection techniques often have negative environmental effects due to the use of chemicals and energy-intensive processes. [14]. The environmental impact assessment of magnetic field applications helps determine its suitability as an environmentally friendly disinfection method [27]. Given the increasing global emphasis on sustainable water treatment solutions and the potential of magnetic field-based technologies, a systematic review is necessary to synthesize existing research findings, identify knowledge gaps, and provide recommendations for future studies. Therefore, the aim of this systematic review was to evaluate the mechanisms, efficacy, and challenges of magnetic field-based technologies for water disinfection.

## 2 Methods

### 2.1 Study design

This systematic review aims to evaluate the mechanisms, efficacy, and challenges associated with magnetic fields-based technologies for water disinfection. A comprehensive approach was employed, adhering to the PRISMA (Preferred Reporting Items for Systematic Reviews and Meta-Analyses) guidelines to ensure transparency and reproducibility throughout the review process. The primary research question guiding this systematic review is: “What are the mechanisms, efficacy, and challenges of magnetic fields-based technologies in the disinfection of water?”

### 2.2 Search strategy

A systematic search of the literature was performed using multiple databases, including Web of Science, PubMed, and Scopus, for studies published in English from January 2010 to March 2025. The search terms employed included “magnetic fields,” “electromagnetic fields,” “water disinfection,” “magnetic water treatment,” “magnetic field technology,” “water purification,” and “efficacy of magnetic fields.” These terms were combined using Boolean operators (AND, OR) to ensure a comprehensive retrieval of relevant literature (Table 1).

### 2.3 Inclusion and exclusion criteria

The inclusion and exclusion criteria in this review have been described in Table 2.

### 2.4 Data extraction

Data extraction was performed using a standardized data extraction form. This form was designed to capture essential study characteristics, such as authorship, year of publication, sample size, methodological approach, magnetic fields application, outcome measures, results, and conclusions. Any discrepancies in data extraction were resolved through discussion and consensus [28].

### 2.5 Quality assessment

The quality of the included studies was assessed using the Critical Appraisal Skills Program (CASP) tool, which consists of 10 questions that address key aspects of study quality. These dimensions include the clarity of the research question, appropriateness of the study design, robustness of data collection methods, rigor of data analysis, and consideration of ethical issues. For this purpose, the checklist of the CASP tool was used to evaluate the quality of qualitative and quantitative studies. The questions were answered “yes”, “to some extent”, “no”, and the authors assigned numbers to each answer as “yes = 2”, “partially = 1”, “no = 0”. Scoring was also used to assess the quality of studies. For qualitative and systematic reviews, a total score of 20 was calculated for each article and graded as high quality (score = 20–16), moderate quality (score = 10–15), or low quality (score = 1–9). This grading system was employed to enhance methodological transparency and provide a clear framework for understanding the reliability of the evidence.

### 2.6 Data synthesis

A narrative synthesis was utilized to summarize the findings from the included studies. Studies were categorized based on the type of magnetic field technology employed, the mechanisms identified, and the reported efficacy in water disinfection. Where appropriate, quantitative data were pooled for meta-analysis, particularly efficacy results expressed in terms of log reduction of microbial contaminants.

### 2.7 Interpretation of results

The results were interpreted in the context of existing literature, highlighting key findings, contradictions, and gaps in knowledge. The implications of magnetic fields-based technologies for practical applications in water disinfection were discussed, considering factors such as scalability, cost-effectiveness, and environmental impact.

### 2.8 Statistical analysis

A meta-analysis was conducted using Comprehensive Meta-Analysis Software (CMA) version 3.0. Effect sizes were calculated as standardized mean differences (SMD) with 95 % confidence intervals (CIs) for continuous outcomes (e.g., gene expression levels). Heterogeneity among studies was assessed using I^2^ statistics, with I^2^ values of 25 %, 50 %, and 75 % representing low, moderate, and high heterogeneity, respectively. A random-effects model was employed for analyses due to expected variability among studies.

### 2.9 Sensitivity analysis and publication bias

Sensitivity analyses were conducted to determine the robustness of the results by excluding studies with high risk of bias. Publication bias was assessed using funnel plot, considering a *p*-value of <0.05 as indicative of significant bias.

### 2.10 Reporting

The findings of this systematic review were reported in alignment with PRISMA guidelines, ensuring comprehensive and transparent presentation of the review process and outcomes (Appendices A and B).

## 3 Results

### 3.1 Description of the included studies

**Fig. 1:**
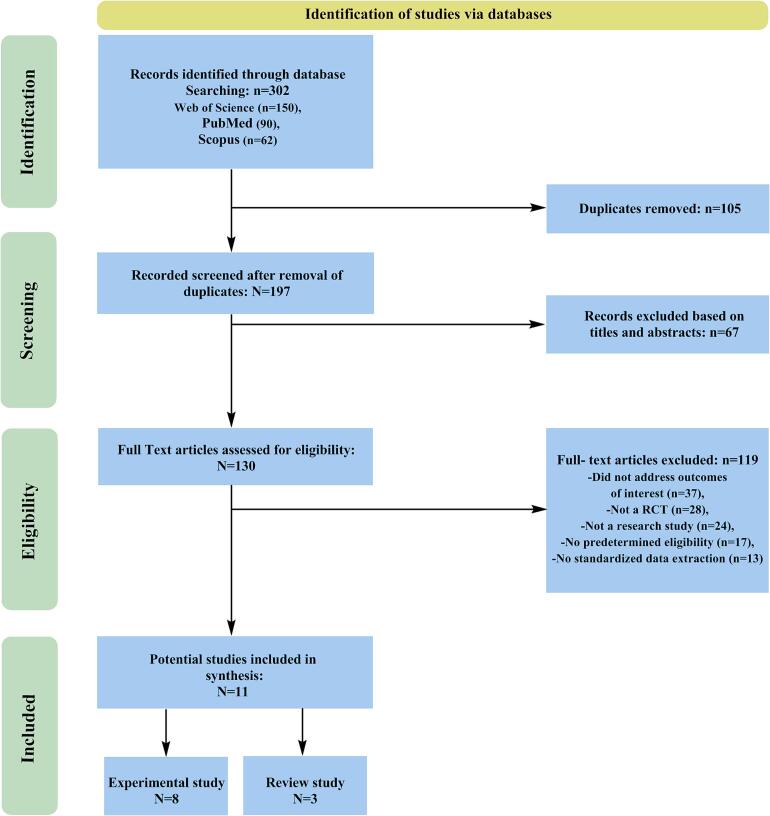

After conducting a thorough search of the selected databases following the methods outlined in the methods section, 11 articles were identified from a total of 302 related studies. The included studies consisted of experimental studies (*n* = 8) and review studies (*n* = 3). The PRISMA flow diagram is presented in Fig. 1, and the characteristics of the studies included in the review are detailed in Table 1.

**Table 1:** Characteristics of the included studies in the present study.

Study No.	First author	Year	Country	Study type	Objective	Study design	Main outcomes
1	Pina al. [20]	2014	Portugal	Experimental	Developing the magnetic antimicrobial agents for water disinfection	The PEG-coated magnetic nanoparticles conjugated with antimicrobial peptide (RW) 3 were synthesized. Then the antimicrobial efficacy against *E. coli* and *Bacillus subtilis* using a high-throughput screening platform to determine MIC via measurement of bacterial growth inhibition was tested. Finally, the bactericidal activity based on MIC values was evaluated.	The developed PEG-coated magnetic nanoparticles conjugated with antimicrobial peptide (RW) 3 demonstrated a MIC of 500 μM against *E. coli* and *B. subtilis*. The nanoparticles effectively disinfected contaminated water, significantly reducing bacterial populations, indicating their potential as nanoscale agents for wastewater disinfection.
2	Santos et al. [29]	2021	Portugal	Experimental	Assessing the disinfection potential of modified magnetic iron oxide nanoparticles	Seven types of magnetic iron oxide nanoparticles modified with compounds like CNTs, copper, and silver were synthesized. Then, the disinfection efficacy against *E. coli* (and other bacteria) by measuring log reductions under varying concentrations and contact times was tested. Moreover, the stability and metal leaching to confirm robustness was assessed	Magnetic iron oxide nanoparticles modified with compounds like copper, silver, and CNTs showed high disinfection efficacy against *E. coli*, with log reductions up to nearly 3. Especially notable was CuFeO/CNT, which achieved approximately 99 % bacterial removal and demonstrated stability with low metal leaching, highlighting their utility in water treatment.
3	Samarghandi et al. [30]	2016	Iran	Experimental	Investigating the magnetic field effects on water microorganisms	The magnetic fields of 100, 200, 300 mV/L using solenoid coils for different exposure times (10–50 min) was applied. Then, water parameters (coliforms, heterotrophic bacteria, turbidity, and pH) before and after exposure under controlled lab conditions to evaluate microbial increase or decrease were monitored.	Magnetic fields produced by electronic devices increased bacterial counts, including coliforms and heterotrophic bacteria, in water samples. The magnetic exposure led to significant increases in bacterial populations, suggesting magnetic fields can promote microbial proliferation in water.
4	Zhang et al. [31]	2017	China	Review	Reviewing the biological effects of static magnetic fields (SMF)	The literature of categorizing effects of SMF on microorganisms, plants, and animals was investigated. Then, the cellular, genetic, and biochemical responses, emphasizing mechanisms and applications related to microbial growth, gene expression, and antibiotic resistance were evaluated.	Static magnetic fields (SMF) exert diverse effects on microorganisms, including alterations in cell growth, morphology, gene expression, and antibiotic resistance. SMFs influence cellular processes, indicating their potential to modulate microbial behavior and applications in biotechnological processes.
5	Guo et al. [17]	2022	China	Review	Summarizing the pulsed magnetic field (PMF) sterilization in food	The literature of existing studies on PMF characteristics, mechanisms, and microbial inactivation efficacy was reviewed. Then, non-thermal sterilization effects, combining PMF with other methods for enhanced microbial reduction were investigated.	Pulsed magnetic field (PMF) technology effectively inactivates pathogens and spoilage microorganisms in food processing, offering a non-thermal sterilization method that preserves food quality while reducing microbial load. It shows promise as a sustainable food sterilization technique.
6	Zaidi et al. [32]	2014	Malaysia	Review	Reviewing the magnetic field applications in water/wastewater treatment	The literature comparing magnetically assisted purification processes with conventional methods was systematically reviewed. Then, physical (particle aggregation) and biological (bacterial activity) effects, and improvements in treatment efficiency were discussed.	Magnetic fields enhance wastewater treatment by improving solid-liquid separation through colloid aggregation and increasing bacterial activity, thereby boosting overall treatment efficiency. Magnetic application shows significant potential to augment conventional water treatment processes.
7	Alkhazan et al. [19]	2010	Saudi Arabia	Experimental	Evaluating the magnetic field effects on lake water quality	Stagnant lake water was treated with magnetic fields of varying intensities under static and turbulent conditions for 30 days. In addition, physical, chemical, and microbiological parameters (clarity, pH, odor, EC, lead ions, bacteria) pre- and post-treatment to assess improvements were measured.	Treating stagnant lake water with magnetic fields improved water clarity, increased pH, reduced odor, electrical conductivity, lead ions, and bacterial content. Magnetic treatment effectively remediated polluted water, demonstrating environmental remediation potential.
8	Kim et al. [33]	2017	USA	Experimental	Controlling the microorganisms via magnetotaxis	*Tetrahymena piriformis* was modified with iron oxide nanoparticles to respond to magnetic fields. Helmholtz coils were used to generate rotating magnetic fields. Swimming behavior, collective movement, and response to magnetic stimuli were observed and characterized.	Magnetically modified *Tetrahymena pyriformis* exhibited controlled swimming behavior under rotating magnetic fields (magnetotaxis). This demonstrates the feasibility of remotely controlling microorganism swarming, with applications in targeted delivery or environmental monitoring.
9	Novickij et al. [34]	2014	Lithuania	Experimental	Enhancing the antifungal efficacy with pulsed magnetic fields	Pathogenic fungi (*Aspergillus, Candida, Trichophyton*) were exposed to microsecond pulsed magnetic fields up to 6.1 Tesla, along with antifungal agents. In addition, fungal viability was assessed to determine synergistic effects.	Combining microsecond pulsed magnetic fields with antifungal agents significantly reduced the viability of pathogenic fungi (*A. fumigatus*, *C. albicans*, *T. rubrum*), indicating a synergistic effect that enhances antifungal efficacy and suggests a potential for improved fungal infection treatments.
10	Liu et al. [22]	2017	China	Experimental	Assessing the magnetic field direction on bacterial sterilization	Heterotrophic bacteria in circulating cooling water were exposed to low-frequency square wave pulsed magnetic fields. Then, the sterilization efficiency with magnetic fields parallel and non-parallel to the flow was compared and the bacterial reduction rate was analyzed.	The electromagnetic pulse sterilization of heterotrophic bacteria in cooling water was more effective when water flow was parallel to the magnetic field, due to interactions with diamagnetic bacterial cell membranes. This indicates flow direction influences sterilization efficiency.
11	Varkey et al. [21]	2018	Swaziland	Experimental	Inactivating *E. coli* with electric and magnetic fields	Contaminated deionized water was treated with moderate AC electric fields (10 V/cm to 1 kV/cm) and static magnetic fields (10–65 mT). Then, the rate of bacterial inactivation after exposure was measured to evaluate its effectiveness for potential low-cost disinfection.	Moderate electric and static magnetic fields achieved up to 90 % inactivation of *E. coli* in deionized water, demonstrating their effectiveness as low-cost, practical disinfection methods suitable for household applications.

### 3.2 Quality assessment of the intended studies

This study followed the CASP tool for assessing the quality of the considered studies. The studies included in this study were experimental studies (*n* = 8) and review studies (*n* = 3). In general, the results of the qualitative assessment of these studies revealed that 9 studies were of high quality and 2 studies were of moderate quality (Table 4).

### 3.3 The meta-analyses of the included studies

**Fig. 2:**
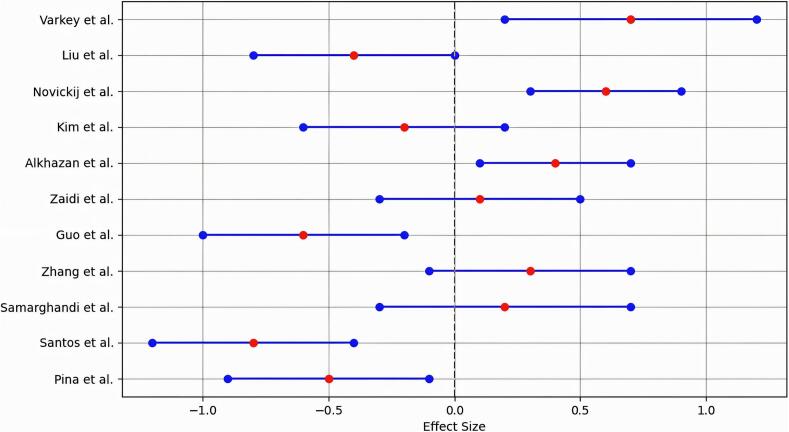
Forest plot of the meta-analyses of the included studies.

**Fig. 3:**
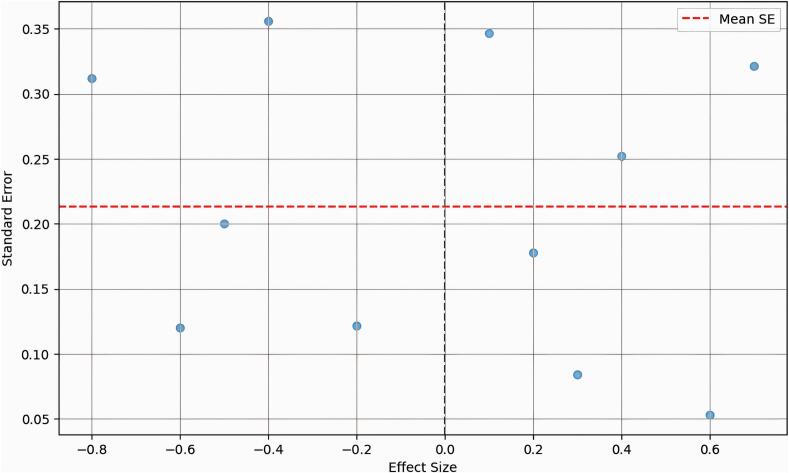
Funnel plot of the meta-analyses of the included studies.

The systematic review and meta-analysis included a total of 11 studies that investigated the effects of magnetic and electromagnetic methods on water disinfection. The overall effect sizes, along with their respective confidence intervals, were calculated to assess the efficacy of these methods in reducing microbial contamination in water. The meta-analysis results**,** forest plot, and funnel plot of the included studies have been provided in Table 5, Fig. 2, Fig. 3, respectively.

The results indicated a mixed efficacy of magnetic and electromagnetic methods in water disinfection. Negative effect sizes were reported in several studies suggesting a potential reduction in disinfection efficacy or ineffective outcomes. Specifically, Santos et al. reported the most substantial negative effect size of −0.8 (CI: −1.2, −0.4), indicating significant concerns over the efficiency of these methods in certain contexts.

Conversely, several studies demonstrated positive effect sizes, indicating potential benefits. Notably, Varkey et al. reported an effect size of 0.7 (CI: 0.2, 1.2), representing a statistically significant improvement in disinfection efficacy. Similarly, Novickij et al. and Alkhazan et al. also found positive effects, with effect sizes of 0.6 and 0.4, respectively.

Overall, the meta-analysis revealed the variability in outcomes across studies, suggesting that while some studies support the effectiveness of magnetic and electromagnetic methods in water disinfection, others raise concerns regarding their efficacy. The presence of both positive and negative effect sizes provided the need for further research and standardization in methodologies to better understand the potential of these innovative disinfection methods.

## 4 Discussion

This systematic review revealed several significant findings regarding their mechanisms, efficacy, and the challenges faced in practical applications. The parameters measured in each of the included studies have been provided in Table 2. The results of the meta-analysis provided a nuanced understanding of the efficacy of magnetic and electromagnetic methods in water disinfection, as evidenced by the varied effect sizes reported across the included studies. The overall trend indicated a mixed and context-dependent effectiveness of these methods, with certain studies indicating significant reductions in microbial load while others report negligible or even counterproductive effects.

**Table 2:** The parameters evaluated in each of the included studies.

Study No.	Measured parameter
1	• Minimum Inhibitory Concentration (MIC) of PEG-coated magnetic nanoparticles against *Escherichia coli* and *Bacillus subtilis*. • Concentration of nanoparticles required to inhibit visible bacterial growth. • Reduction in bacterial populations in solution (quantitative analysis of bacterial counts).
2	• Synthesis and modification of magnetic iron oxide nanoparticles. • Concentration of magnetic nanoparticles (50 mg/mL). • Contact time for disinfection (1 min). • Log reduction values and removal efficiency for *Escherichia coli* and *Staphylococcus aureus*. • Bacterial efficiency in single and mixed bacterial suspensions.
3	• Magnetic field intensities (100, 200, and 300 mV/L). • Exposure times (10, 20, 30, 40, and 50 min). • Water sample parameters: temperature, pH, turbidity, total coliforms, fecal coliforms, plate count of heterotrophic bacteria. • Most Probable Number (MPN) of coliforms and heterotrophic bacteria.
4	• Categories of effects of Static Magnetic Fields (SMF) on microorganisms: cell growth and viability, morphological and biochemical changes, genotoxicity, gene and protein expression, magnetosome formation, antibiotic resistance, fermentation, and wastewater treatment. • Variations in response based on magnetic field intensities.
5	• Characteristics and operating principles of pulsed magnetic field (PMF) technology. • Biological effects of PMF on microorganisms. • Comparison of PMF with traditional heat treatment regarding food quality and safety.
6	• Applications of magnetic fields in wastewater treatment. • Performance metrics compared to conventional treatment methods (e.g., solid-liquid separation, bacterial activity). • Environmental benefits of magnetically assisted treatment.
7	• Magnetic field intensities (static and agitated conditions). • Treatment duration (30 days). • Physical and chemical properties: water clarity, pH, odor, electrical conductivity (EC), lead ion concentration, bacterial content.
8	• Modification of *Tetrahymena pyriformis* using iron oxide nanoparticles. • Swimming behavior in response to rotating magnetic fields. • Characteristics of the rotating magnetic fields.
9	• Pulsed magnetic field intensities (up to 6.1 T). • Viability of pathogenic fungi (*Aspergillus fumigatus, Candida albicans, Trichophyton rubrum*) after treatment.
10	• Low-frequency square wave pulse magnetic fields. • Water flow direction (parallel and antiparallel to the magnetic field). • Sterilization efficiency.
11	• Moderate AC electric fields (10 V/cm to 1 kV/cm). • Static magnetic fields (10 to 65 mT). • Inactivation rate of *E. coli* after exposure to electric and magnetic fields.

While our findings revealed significant differences in effect sizes, ranging from −0.8 to 0.7, the analysis of the underlying causes of heterogeneity was relatively superficial. This limitation demonstrated the need for a more in-depth exploration of factors that may influence the efficacy of these disinfection methods.

One of the primary sources of heterogeneity could be attributed to the varying parameters of the magnetic fields applied in different studies. Factors such as field strength, frequency, and exposure duration can significantly impact the effectiveness of magnetic and electromagnetic disinfection [35]. For instance, higher magnetic field strengths may enhance the generation of reactive species, leading to improved disinfection outcomes. Conversely, suboptimal field parameters could result in diminished efficacy or even negative effects, as indicated by the negative effect sizes observed in some studies [5].

Additionally, the quality of water used in the experiments is another critical factor that warrants further investigation. Variations in water composition, including the presence of organic matter, turbidity, and microbial load, can influence the interaction between magnetic fields and microbial contaminants [6]. For example, higher levels of organic matter may shield microorganisms from the effects of magnetic fields, thereby reducing disinfection efficacy. A subgroup analysis focusing on these parameters could provide valuable insights into how different conditions affect the performance of magnetic and electromagnetic methods [17].

### 4.1 Effect on the various microorganisms

The review demonstrated that the magnetic and electromagnetic fields have various effects on various microorganisms in water, including bacteria, viruses, parasites, and algae and cyanobacteria.

#### 4.1.1 Bacteria

Magnetic fields have been shown to influence the growth and viability of bacteria. Numerous studies indicate that exposing water contaminated with bacteria to magnetic fields can inhibit bacterial growth or lead to bacterial cell death [36]. It is thought that the mechanism of action is related to the disruption of the bacterial cell membrane, changing their permeability and causing structural damage. Various studies have been conducted to investigate the effect of magnetic fields on water bacteria [37].

The exposure to a magnetic field can prevent the growth of some water bacteria. According to studies exposure to a 0.025 T magnetic field for 60 min significantly reduced the growth of *E. coli* bacteria in water samples [38]. It was observed that the magnetic field disrupts the ability of bacteria to divide and reproduce, which leads to a decrease in their number [39].

The magnetic fields cause changes in the metabolic activity of bacteria. A study conducted by researchers at the Indian Institute of Technology found that exposure to a 1mT magnetic field for 24 h changed the enzymatic activity of *Staphylococcus aureus* [40,41]. The studies demonstrated that changes in the production of specific enzymes involved in bacterial metabolism, indicating that magnetic fields can affect the metabolic processes of water bacteria [40].

In a study, László et al. investigated the effect of static magnetic fields on the microorganisms *Saccharomyces cerevisiae, Bacillus circulans, Escherichia coli, Micrococcus luteus, Pseudomonas fluorescens, Salmonella enteritidis, Serratia marscens* and *Staphylococcus aureus*. In this study, these microorganisms were exposed to three types of static magnetic fields: homogeneous, heterogeneous, and with a lateral magnetic flux density gradient. The results of their study showed that even the longest period of exposure to these three types of magnetic fields had no effect on the growth of bacteria [36].

Konopacki et al. also evaluated the effect of rotating magnetic field on the growth rate and cellular metabolic activity of Gram-positive bacteria (*Staphylococcus aureus, Enterococcus faecalis and Streptococcus*) and Gram-negative bacteria (*Escherichia coli, Serratia marscens and Klebsiella oxytoca*). In their study, the liquid cultures of these bacteria were exposed to a rotating magnetic field with a frequency of f = 50 Hz and a magnetic induction of Bmax = 18 mT for 8 h at a temperature of 37 °C. The results of their study showed that magnetic fields increased the metabolic activity of Gram-positive bacteria (up to 25 %) and inhibited the proliferation of Gram-negative bacteria (up to 17 %). Based on their results, it can be concluded that rotating magnetic fields affect the cell wall transfer mechanisms and this effect is different for these two groups of bacteria. In general, magnetic fields have a positive effect on gram-positive bacteria and a negative effect on gram-negative bacteria in terms of antimicrobial properties [42].

Similarly, Fijałkowski et al. the effects of rotating magnetic field (RMF) on growth, cellular metabolic activity and biofilm formation of *Staphylococcus aureus, E. coli, A. baumannii, P. aeruginosa, S. marcescens, S. mutans, C. sakazakii, K. oxytoca* and *S. xylosus*. The microorganisms were exposed to RMF (magnetic induction RMF B = 25–34 mT, RMF frequency f = 5–50 Hz, exposure time *t* = 60 min, incubation temperature 37 °C). The persistence of the effect of exposure (B = 34 mT, f = 50 Hz, *t* = 60 min) on bacteria after further incubation (*t* = 300 min) was also studied. The work showed that exposure to RMF stimulated the investigated parameters of *Staphylococcus aureus, E. coli, S. marcescens, S. mutans, C. sakazakii, K. oxytoca and S. xylosus*, but cellular metabolic activity and biofilm formation is restrained by *A. bomani* and *P. aeruginosa*. The results obtained from their study proved that RMF can modulate the functional parameters of different bacterial species depending on its magnetic induction and frequency [43].

It is important to note that more research is needed to fully understand the mechanisms and potential applications of magnetic fields in controlling water bacteria. Effects can vary depending on the specific characteristics of the magnetic field (e.g., strength, duration of exposure) and the type of bacteria being studied [44].

#### 4.1.2 Viruses

Magnetic fields have demonstrated inhibitory effects on specific types of viruses. For instance, studies indicate that exposure to magnetic fields can decrease infections caused by the hepatitis A virus and the poliovirus [10]. Magnetic fields are thought to interfere with virus replication, genome stability, and protein synthesis. While the effects of magnetic fields on water viruses in particular have not been extensively studied, some research suggests that magnetic fields can have an inhibitory or stimulatory effect on virus activity and replication in water [45].

In one study, researchers found that exposure to a specific pattern of magnetic field significantly inhibited the proliferation of certain viruses in an aquatic environment. The magnetic field disrupted the structure of viral particles or affected their interaction with water molecules, leading to a decrease in viral load [46]. This suggests that magnetic fields could potentially be used as a method to control waterborne viral infections. In addition, there are also studies showing that certain types of magnetic fields can stimulate viral activity [47]. For example, a study on the effect of magnetic fields on bacteriophages (viruses that infect bacteria) showed that exposure to a certain frequency of the magnetic field increased bacteriophage infectivity. This provides that magnetic fields can affect the binding and entry of viruses into host cells, potentially increasing their replication. It is important to note that the effect of magnetic fields on water viruses can vary depending on various factors such as specific virus species, magnetic field intensity and frequency, and general environmental conditions [48]. Further research is needed to better understand the mechanisms through which magnetic fields interact with water viruses and the potential applications in water treatment and virus control.

#### 4.1.3 Parasites

Magnetic fields have also been investigated for their potential in targeting aquatic parasites. Studies have shown that exposure to magnetic fields can have a detrimental effect on the viability and infectivity of parasites such as *Giardia* and *Cryptosporidium* [49]. Magnetic fields may affect the cell membrane, respiration and metabolic processes of the parasites and lead to their inactivation. This effect is primarily through disruption of their vital functions and activities, which ultimately leads to disruption of their growth, reproduction and survival [50].

Caliskan et al. examined the effect of magnetic fields on the parasite *Toxoplasma gondii* showed that exposure to a 50 Hz magnetic field resulted in reduced parasite infection [51]. The study attributed these effects to changes in the parasite's protein metabolism and disruption of its ion channels. Fagundes Teixeira et al. also focused on the effect of magnetic field on the larval stage of *Schistosoma mansoni* parasite, which causes schistosomiasis. It was observed that a magnetic field with a frequency of 30 Hz and an intensity of 2 mT led to a significant decrease in parasite viability and infectivity [52]. The magnetic field interferes with the parasite's neuromuscular function, disrupting its motility and ability to infect the host. In addition, magnetic fields have disturbed the orientation and movement of various water parasites [53].

In the case of the parasite *Lepeophtheirus salmonis* that infects salmon, exposure to a 0.65 mT magnetic field alters the parasite's ability to locate and attach to host fish. The presence of the magnetic field disrupted the parasite's magnetic and navigational systems and made it challenging for the parasite to parasitize the host [54]. However, it is important to note that the effect of magnetic fields on aquatic parasites can vary depending on the specific parasite, field intensity, frequency, and duration of exposure. In addition, the effect of magnetic fields on other organisms or the environment should also be considered when evaluating their potential as a control method for aquatic parasites [48].

### 4.2 Efficacy in water treatment and disinfection

Moreover, the included studies demonstrated a diverse range of methodologies, materials, and outcomes, highlighting the multifaceted nature of magnetic field applications in water treatment. Some key applications and concepts related to the use of magnetic fields in reducing waterborne microorganisms have been described in Table 3.

**Table 3:** Some major applications of magnetic and electromagnetic fields in reducing waterborne microorganisms [18,55].

No.	Application	Explanation
1	Magnetically induced flocculation	• Mechanism: Certain magnetic materials can be added to water, which then respond to magnetic fields. When exposed to a magnetic field, these materials can agglomerate microorganisms and other contaminants, forming larger flocs that can be more easily removed from the water. • Applications: This method is particularly useful in wastewater treatment and in the removal of specific pathogens.
2	Magnetic separation	• Mechanism: Magnetic separation involves the use of magnetic fields to extract magnetic particles from a liquid medium. In water treatment, magnetic nanoparticles can be engineered to bind to specific microorganisms. Once attached, these particles can be separated from the water using a magnetic field. • Applications: This technique is effective for removing bacteria, viruses, and protozoa from contaminated water.
3	Electromagnetic fields and cell disruption	• Mechanism: Electromagnetic fields can affect microbial cells by disrupting their membrane integrity, leading to cell lysis. This phenomenon can be enhanced when using pulsed electromagnetic fields (PEMFs), which have been shown to increase the permeability of cell membranes. • Applications: This approach can be used for the inactivation of pathogens in water, making it a potential method for improving drinking water safety.
4	Magnetic nanoparticles for antimicrobial delivery	• Mechanism: Magnetic nanoparticles can be functionalized with antimicrobial agents. When these nanoparticles are exposed to a magnetic field, they can be directed to specific locations in water, allowing for targeted delivery of the antimicrobial agents to microbial communities. • Applications: This method can enhance the effectiveness of disinfection processes by concentrating the antimicrobial agents where they are needed most.
5	Magnetohydrodynamics (MHD)	• Mechanism: MHD refers to the behavior of electrically conducting fluids in the presence of magnetic fields. This principle can be applied to enhance the mixing and flow of water, which can improve the efficacy of disinfection processes (e.g., chlorine or UV treatment). • Applications: MHD can be integrated into existing water treatment systems to optimize the contact time between disinfectants and microorganisms.
6	Biofilm control	• Mechanism: Magnetic fields may disrupt biofilms, which are clusters of microorganisms adhering to surfaces. • Applications: The application of a magnetic field can inhibit biofilm.

The efficacy of magnetic field technologies extends beyond nanoparticles. For instance, studies such as those by Alkhazan et al. and Varkey et al. indicated that both static and alternating magnetic fields can lead to significant improvements in water quality, including reductions in bacterial content and enhancements in physical properties such as clarity and pH. These findings showed the potential of magnetic field applications as complementary technologies in traditional water treatment systems [19,21].

Zhang et al. provided a broader perspective by categorizing the effects of static magnetic fields on various organisms, emphasizing their diverse biological impacts [31]. The review showed that magnetic fields can influence cell viability, growth patterns, and even antibiotic resistance in microorganisms, suggesting that their application in water treatment could extend to mitigating resistance issues and enhancing overall microbial management.

### 4.3 Mechanisms of action

The included studies revealed that magnetic and electromagnetic fields can affect various biological and chemical processes, and their application in water disinfection technologies has attracted attention due to their potential to inactivate pathogens without the use of harsh chemicals. The underlying mechanisms by which magnetic fields and associated nanotechnologies exert their antimicrobial effects are critical to understanding their potential for water disinfection. For instance, Pina et al. demonstrated that PEG-coated magnetic nanoparticles conjugated with antimicrobial peptides could significantly reduce bacterial populations in contaminated water. The minimum inhibitory concentration (MIC) of these nanoparticles was established at 500 μM for both *Escherichia coli* and *Bacillus subtilis*, indicating their efficacy in targeting both Gram-positive and Gram-negative bacteria [20].

Similarly, Santos et al. evaluated various modifications of magnetic iron oxide nanoparticles, revealing that those modified with copper and carbon nanotubes exhibited exceptional disinfection efficiency against *E. coli*, achieving log reductions of nearly 3.0. This shows that the functionalization of magnetic nanoparticles can enhance their interaction with bacterial cells, leading to improved antimicrobial action [29].

Moreover, the study by Samarghandi et al. indicated that exposure to magnetic fields can significantly increase bacterial counts in certain scenarios, thus highlighting the need for careful consideration of magnetic field parameters and their potential biological impacts [30].

**Fig. 4:**
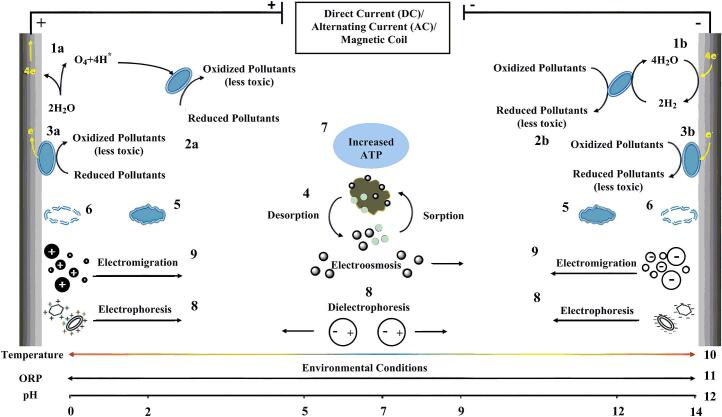
Effects of electric, magnetic and electromagnetic fields on microorganisms. (1) Hydrolysis leading to the production of O_2_ (a) and the production of H_2_ (b), (2) Partial oxidation (a) / reduction (b) of pollutants, (3) Solid electrodes as electron acceptors (a) / donors (b), (4) Increase in pollutant bioavailability, (5) Modification in cell physiology and morphology, (6) Loss of membrane integrity, with release of cytoplasmic materials and cell death, (7) Increase in intracellular ATP concentration, (8) Increased transport of organic molecules, nutrients and bacterial cells due to electroosmosis, electrophoresis and dielectrophoresis, (9) Transport of dissolved ions due to electromigration, (10) Increase in temperature near the electrodes, (11) Divergence of redox potential from ambient conditions, (12) Change in pH near the electrodes [57].

There are the main mechanisms of action through which magnetic fields may aid in water disinfection. The effects of electric, magnetic and electromagnetic fields on microorganisms have been illustrated in Fig. 4. Magnetic fields can enhance electrochemical reactions, leading to the production of reactive oxygen species (ROS) when water is exposed to an electric current. These ROS can effectively inactivate bacteria and viruses [56].

ROS are highly reactive oxygen-containing molecules produced as natural byproducts of cellular metabolism. They play essential roles in various biological processes, including cell signaling, immune response, and regulation of cellular functions [58]. However, excessive ROS levels can lead to oxidative stress, damaging cellular components like DNA, proteins, and lipids, which may result in diseases such as cancer, cardiovascular diseases, and neurodegenerative disorders. The main types of ROS include superoxide anion (O^2•−^), hydrogen peroxide (H_2_O_2_), Hydroxyl Radical (^•^OH), and Singlet Oxygen (^1^O_2_) [59,60].

Li et al. investigated how static magnetic fields (SMFs) affect bacterial growth through the mediation of ROS, focusing on *E. coli*. The results showed that exposure to a SMFs at 250 mT significantly increased ROS levels, which was confirmed by various methods including chemical fluorescent probe and electron paramagnetic resonance (EPR) spectroscopy. Transcriptomic analysis revealed that SMF and hydrogen peroxide (H_2_O_2_) treatment shared differentially expressed genes (DEGs) related to long-chain fatty acid metabolism, the tricarboxylic acid (TCA) cycle, and ROS defense mechanisms. Notably, SMFs downregulated the fadD gene that is essential for the degradation of long-chain fatty acids, thereby impairing bacterial growth. Furthermore, overexpression of the superoxide dismutase gene SodB attenuates SMFs-induced growth inhibition, highlighting the critical role of ROS in this process [61]. Overall, the findings elucidate the molecular mechanisms by which oxygen acts as a magnetic target, initiates ROS signaling, and allows bacteria to adapt to SMF exposure.

The use of magnetic fields also can induce mechanical stress on microbial cell membranes. This stress may disrupt the integrity of the membranes, leading to cell lysis and death [50].

Oncul et al. reported that the low-frequency electromagnetic field (ELF-EMF) changed in the physicochemical properties of both Gram-positive and Gram-negative bacteria. Hyperpolarization was observed in *S. aureus* and *E. coli* treated with EDTA. The surface potential in *S. aureus* showed a positive change, in contrast to the negative change observed in *E. coli* not treated with EDTA. Respiratory activity increased in both bacteria. A slight decrease in growth was observed [62].

Furthermore, magnetic fields can increase the mixing and mass transfer of water and improve contact between pathogens and disinfectants (such as chlorine or ozone) that may be present in the water [63]. The interaction of magnetic fields with conductive materials in water can also induce electric currents that potentially lead to localized heating and the production of free radicals that can damage microbial cells [64].

Moreover, magnetic fields may affect the orientation and movement of charged particles, ions, and water molecules, which can affect biochemical processes in microorganisms and disrupt their metabolic function [65]. Besides, magnetic fields can affect the metabolic pathways of bacteria, potentially inhibiting growth and reproduction. This may be due to changes in enzymatic activity or disruption of ATP production [66]. Additionally, some microorganisms may aggregate together in the presence of magnetic fields, which can enhance their removal from water through sedimentation or filtration processes [67].

Some studies demonstrated that magnetic fields can alter the physical properties of water, including its structure and hydrogen bonding, which may increase the solubility and reactivity of disinfectants [68]. These mechanisms can work independently or synergistically, depending on the specific conditions of the water treatment process and the characteristics of the target pathogens [69,70].

A water molecule is a polar molecule because it has a positive charge at one end and a negative charge at the other due to the sharing of electrons at one end. The effect of a magnetic field on a water molecule is explained by this polarity [32]. Polar molecules can be positioned in any direction in the absence of a magnetic field. Therefore, when molecules collide, their positive and negative charges repel each other. On the other hand, polar molecules easily align based on their positive and negative charges when exposed to a magnetic field of with certain strength [8].

Recent studies have shown that an external magnetic field induces Lorentz forces on water molecules, leading to their interaction, resulting in the magnetization of liquid water and a potential effect on water clusters [71]. It has been observed that the effect of magnetization on water is attributed to a change in the molecular energy of water molecules. Furthermore, changes in the average size of water clusters have been shown after exposure to magnetization. The interaction between an external magnetic field and water molecules can lead to significant changes in the properties of water, potentially affecting its behavior and structure [18].

## 5 Contributions to the One Health framework

The investigation of magnetic and electromagnetic methods for water disinfection offers significant environmental benefits compared to traditional chemical disinfectants. One of the main benefits is the reduction of harmful chemical by-products that often result from conventional disinfection processes. By minimizing reliance on chemicals, these innovative methods align with the One Health goals that emphasize the importance of reducing environmental damage and promoting holistic health in human, animal, and environmental systems. This approach not only protects water quality, but also contributes to the sustainability of ecosystems and fosters healthier environments [72].

Improving water disinfection through these methods has the potential to significantly reduce the burden of waterborne diseases, which pose a serious threat to public health. By effectively controlling microbial contamination, these technologies can reduce the incidence of diseases such as cholera, dysentery, and typhoid fever. Furthermore, since many waterborne pathogens are shared between humans and livestock, improving water quality can also reduce the transmission of diseases from animals to humans, thereby improving the overall health and safety of the community [73].

Interdisciplinary collaboration is essential to realize the full potential of magnetic and electromagnetic disinfection methods. Collaboration with ecologists, veterinarians, and public health professionals can increase understanding of how these technologies can be optimized for diverse applications [74]. For example, ecologists can provide insights into the environmental impacts of these methods, while veterinarians can help assess their effectiveness in controlling pathogens shared between humans and livestock. Public health professionals can provide valuable knowledge about the implications for community health and disease prevention. Such collaborations can lead to more comprehensive and effective water management strategies that incorporate the benefits of these innovative disinfection technologies [75].

This study contributed to the One Health framework by demonstrating how magnetic and electromagnetic methods can provide sustainable solutions for water disinfection. These methods are aligned with the overall One Health goal of promoting the interconnectedness of human, animal, and environmental health by supporting healthier ecosystems and communities. The findings of this meta-analysis can inform policies and practices that incorporate One Health principles and support comprehensive water management strategies that consider the health of all living systems. By integrating these innovative disinfection methods into policy frameworks, stakeholders can improve public health outcomes while protecting the environment.

While the findings indicated promising disinfection efficacy at the laboratory scale, it is essential to consider the implications for real-world water treatment systems. Most studies were conducted under controlled conditions, which may not reflect the complexities of large-scale applications.

Key considerations for practical implementation include energy consumption, maintenance costs, and operational feasibility. The energy demands for maintaining effective magnetic or electromagnetic fields in larger operations could be significant, necessitating further research to compare these methods with traditional disinfection techniques like chlorination or UV treatment. Additionally, the durability and reliability of the equipment in real-world conditions are crucial for assessing long-term operational costs.

Scalability is another important factor, as the effectiveness of these methods may vary based on water quality, flow rates, and microbial populations. In summary, while the meta-analysis revealed the potential of these innovative disinfection technologies, further research is required to evaluate their applicability in real-world settings, focusing on energy consumption, maintenance, and scalability to facilitate their integration into existing water treatment frameworks.

In conclusion, magnetic and electromagnetic fields-based technologies for water disinfection represent a promising frontier in water treatment solutions. The included studies revealed considerable potential for enhanced disinfection efficacy through the use of magnetic nanoparticles and exposure to magnetic fields. However, further research is essential to fully understand the mechanisms involved, optimize parameters for real-world applications, and address potential challenges associated with their implementation. Continued exploration in this field could lead to innovative and sustainable solutions for water disinfection, ultimately contributing to improved public health and environmental sustainability.

## 6 Conclusion

Based on the comprehensive review of current studies, magnetic and electromagnetic methods revealed considerable potential in reducing microbial contamination in water. The application of magnetic nanoparticles functionalized with antimicrobial agents, such as PEG-coated magnetic nanoparticles conjugated with peptides or modified with metals like copper and silver, has shown high efficacy in inactivating bacteria such as *Escherichia coli* and *Bacillus subtilis*. These nanomaterials facilitate effective disinfection through direct antimicrobial action and aggregation processes, offering promising nanoscale solutions for water treatment.

Nanoparticles can enhance antimicrobial properties due to their large surface area, but their residual toxicity poses potential risks to aquatic ecosystems and human health. The toxicity varies based on factors such as composition, size, and surface modifications, necessitating thorough evaluations of their environmental impact. Challenges also arise in recycling or removing nanoparticles from water treatment systems, as their presence complicates the treatment process compared to traditional methods. Developing effective strategies for recovering and recycling nanoparticles is essential to minimize waste and contamination.

Furthermore, the application of static and pulsed magnetic fields has yielded mixed but insightful results. Certain studies indicate that magnetic fields, especially at higher intensities and with agitation, can improve water clarity, reduce chemical contaminants, and diminish bacterial loads, highlighting their utility in environmental remediation. Conversely, some investigations reveal that magnetic fields may inadvertently increase bacterial proliferation, suggesting that magnetic exposure parameters require careful optimization to avoid unintended effects. Emerging technologies such as pulsed magnetic fields and magnetotaxis of microorganisms also show promise for enhancing sterilization efficiency and controlling microbial behavior, especially when combined with conventional antifungal agents or in targeted applications like industrial cooling systems. Additionally, magnetic fields have been noted to improve physical water properties and facilitate biological treatment processes, thus contributing to overall water quality improvements. This study emphasized the importance of integrating innovative disinfection methods into comprehensive water management strategies that aligns with One Health principles, ultimately supporting healthier ecosystems and communities.

## CRediT authorship contribution statement

**Shiva Shahveh:** Methodology, Investigation. **Fatemeh Razaghi:** Writing – original draft. **Maziar Naderi:** Writing – review & editing, Visualization, Validation, Supervision, Project administration, Conceptualization.

## Declaration of generative AI and AI-assisted technologies in the writing process

During the preparation of this work the author(s) used [Talk with ChatGPT] in order to improve readability and language. After using this tool/service, the author(s) reviewed and edited the content as needed and take(s) full responsibility for the content of the published article.

## Declaration of competing interest

The authors declare that they have no known competing financial interests or personal relationships that could have appeared to influence the work reported in this paper.

## Data Availability

Data will be made available on request.

## Appendix A Supplementary data

Supplementary material 2

Supplementary material 1Supplementary material 1
