# Supplementary material for: Magnetic and electromagnetic methods in reducing microbial contamination in water: A systematic review and meta-analyses
Source: One Health. 2025 Sep 20;21:101213. doi: 10.1016/j.onehlt.2025.101213 (PMC12508911; doi:10.1016/j.onehlt.2025.101213)
Supplement: Supplementary file 1 [file mmc1.docx]

**Fig. 1)** PRISMA flow diagram


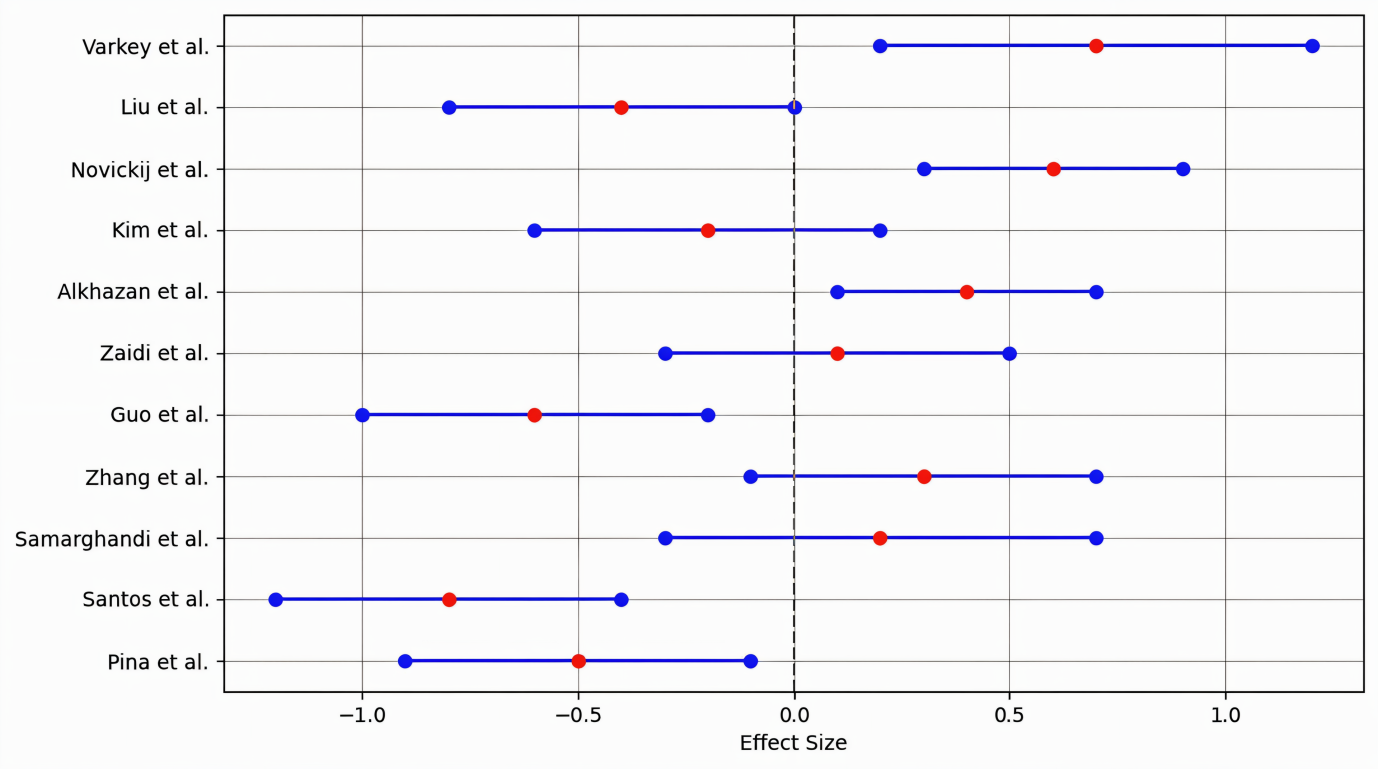


**Fig. 2)** Forest plot of the meta-analyses of the included studies


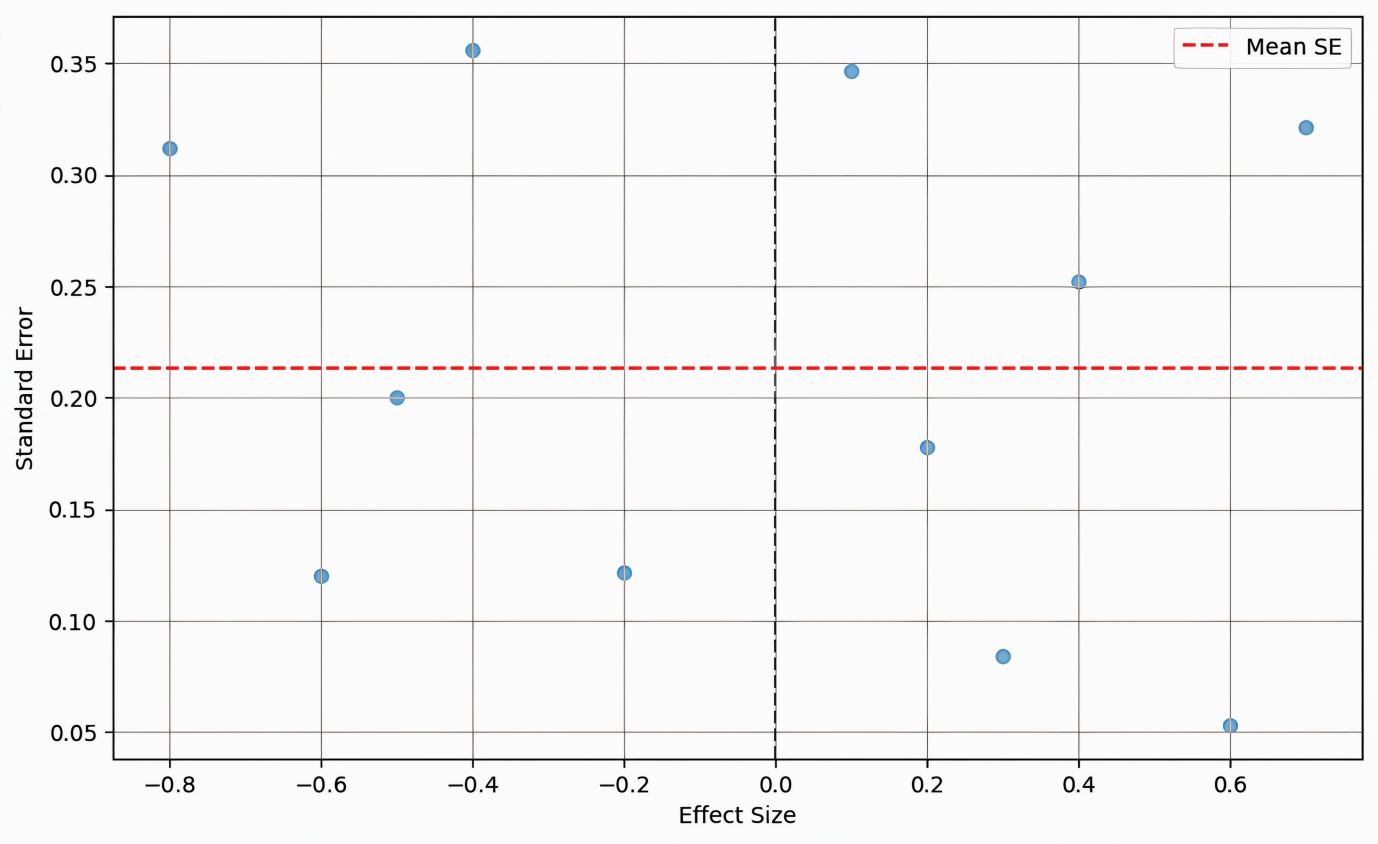


**Fig. 3)** Funnel plot of the meta-analyses of the included studies





**Fig. 4)** Effects of electric, magnetic and electromagnetic fields on microorganisms. (1) Hydrolysis leading to the production of O_2_ (a) and the production of H_2_ (b), (2) Partial oxidation (a) / reduction (b) of pollutants, (3) Solid electrodes as electron acceptors (a) / donors (b), (4) Increase in pollutant bioavailability, (5) Modification in cell physiology and morphology, (6) Loss of membrane integrity, with release of cytoplasmic materials and cell death, (7) Increase in intracellular ATP concentration, (8) Increased transport of organic molecules, nutrients and bacterial cells due to electroosmosis, electrophoresis and dielectrophoresis, (9) Transport of dissolved ions due to electromigration, (10) Increase in temperature near the electrodes, (11) Divergence of redox potential from ambient conditions, (12) Change in pH near the electrodes [[73](#_ENREF_73)].
