# Supplementary material for: Magnetic and electromagnetic methods in reducing microbial contamination in water: A systematic review and meta-analyses
Source: One Health. 2025 Sep 20;21:101213. doi: 10.1016/j.onehlt.2025.101213 (PMC12508911; doi:10.1016/j.onehlt.2025.101213)
Supplement: Supplementary file 2 [file mmc2.docx]

**Table 1)** Search strategy in the considered databases

| **Database** | **Search strategy and possible combination** | **Number of articles retrieved** |
| --- | --- | --- |
|  |  |  |
| **Web of Science** | ("magnetic fields" OR "electromagnetic fields") AND TS=("water disinfection" OR "water treatment") AND TS=(efficacy OR mechanism OR challenges OR technology) | 150 |
| **PubMed** | ("magnetic fields"[MeSH Terms] OR "electromagnetic fields" OR "magnetic field"[All Fields]) AND ("water disinfection"[All Fields] OR "water treatment"[All Fields]) AND (efficacy[All Fields] OR mechanism[All Fields] OR challenges[All Fields] OR technology[All Fields]) | 90 |
| **Scopus** | TITLE-ABS-KEY(("magnetic fields" OR "electromagnetic fields") AND ("water disinfection" OR "water treatment") AND (efficacy OR mechanism OR challenges OR technology)) | 62 |

**Table 2)** Inclusion and exclusion criteria in the present study

| **Inclusion criteria** | **Exclusion criteria** |
| --- | --- |
|  |  |
| Peer-reviewed journal articles, conference papers, and theses related to the application of magnetic and electromagnetic fields in water disinfection. | Non-peer-reviewed articles, opinion pieces, editorials, and commentary. |
| Studies that focused specifically on the efficacy of magnetic and electromagnetic fields in water disinfection. | Studies that did not specifically focus on the effectiveness of magnetic and electromagnetic fields in water disinfection. |
| Studies that have examined the use of magnetic and electromagnetic fields in water disinfection alone. | Studies that have investigated the use of magnetic and electromagnetic fields in water disinfection, along with other disinfection methods such as ultraviolet light (UV) and chemical disinfection. |
| Studies published from January 2010 to March 2025. | Studies published before January 2010. |
| Articles published in English or with available English translations. | Articles not published in English or without available English translations. |

**Table 3)** Characteristics of the included studies in the present study

| **Study No.** | **First author** | **Year** | **Country** | **Study type** | **Objective** | **Study design** | **Main outcomes** |
| --- | --- | --- | --- | --- | --- | --- | --- |
|  |  |  |  |  |  |  |  |
| 1 | Pina al. [[20](#_ENREF_20)] | 2014 | Portugal | Experimental | Developing the magnetic antimicrobial agents for water disinfection | The PEG-coated magnetic nanoparticles conjugated with antimicrobial peptide (RW) 3 were synthesized. Then the antimicrobial efficacy against *E. coli* and *Bacillus subtilis* using a high-throughput screening platform to determine MIC via measurement of bacterial growth inhibition was tested. Finally, the bactericidal activity based on MIC values was evaluated. | The developed PEG-coated magnetic nanoparticles conjugated with antimicrobial peptide (RW) 3 demonstrated a MIC of 500 μM against E. coli and B. subtilis. The nanoparticles effectively disinfected contaminated water, significantly reducing bacterial populations, indicating their potential as nanoscale agents for wastewater disinfection. |
| 2 | Santos et al. [[30](#_ENREF_30)] | 2021 | Portugal | Experimental | Assessing the disinfection potential of modified magnetic iron oxide nanoparticles | Seven types of magnetic iron oxide nanoparticles modified with compounds like CNTs, copper, and silver were synthesized. Then, the disinfection efficacy against *E. coli* (and other bacteria) by measuring log reductions under varying concentrations and contact times was tested. Moreover, the stability and metal leaching to confirm robustness was assessed | Magnetic iron oxide nanoparticles modified with compounds like copper, silver, and CNTs showed high disinfection efficacy against E. coli, with log reductions up to nearly 3. Especially notable was CuFeO/CNT, which achieved approximately 99% bacterial removal and demonstrated stability with low metal leaching, highlighting their utility in water treatment. |
| 3 | Samarghandi et al. [[31](#_ENREF_31)] | 2016 | Iran | Experimental | Investigating the magnetic field effects on water microorganisms | The magnetic fields of 100, 200, 300 mV/L using solenoid coils for different exposure times (10-50 min) was applied. Then, water parameters (coliforms, heterotrophic bacteria, turbidity, and pH) before and after exposure under controlled lab conditions to evaluate microbial increase or decrease were monitored. | Magnetic fields produced by electronic devices increased bacterial counts, including coliforms and heterotrophic bacteria, in water samples. The magnetic exposure led to significant increases in bacterial populations, suggesting magnetic fields can promote microbial proliferation in water. |
| 4 | Zhang et al. [[32](#_ENREF_32)] | 2017 | China | Review | Reviewing the biological effects of static magnetic fields (SMF) | The literature of categorizing effects of SMF on microorganisms, plants, and animals was investigated. Then, the cellular, genetic, and biochemical responses, emphasizing mechanisms and applications related to microbial growth, gene expression, and antibiotic resistance were evaluated. | Static magnetic fields (SMF) exert diverse effects on microorganisms, including alterations in cell growth, morphology, gene expression, and antibiotic resistance. SMFs influence cellular processes, indicating their potential to modulate microbial behavior and applications in biotechnological processes. |
| 5 | Guo et al. [[17](#_ENREF_17)] | 2022 | China | Review | Summarizing the pulsed magnetic field (PMF) sterilization in food | The literature of existing studies on PMF characteristics, mechanisms, and microbial inactivation efficacy was reviewed. Then, non-thermal sterilization effects, combining PMF with other methods for enhanced microbial reduction were investigated. | Pulsed magnetic field (PMF) technology effectively inactivates pathogens and spoilage microorganisms in food processing, offering a non-thermal sterilization method that preserves food quality while reducing microbial load. It shows promise as a sustainable food sterilization technique. |
| 6 | Zaidi et al. [[33](#_ENREF_33)] | 2014 | Malaysia | Review | Reviewing the magnetic field applications in water/wastewater treatment | The literature comparing magnetically assisted purification processes with conventional methods was systematically reviewed. Then, physical (particle aggregation) and biological (bacterial activity) effects, and improvements in treatment efficiency were discussed. | Magnetic fields enhance wastewater treatment by improving solid-liquid separation through colloid aggregation and increasing bacterial activity, thereby boosting overall treatment efficiency. Magnetic application shows significant potential to augment conventional water treatment processes. |
| 7 | Alkhazan et al. [[34](#_ENREF_34)] | 2010 | Saudi Arabia | Experimental | Evaluating the magnetic field effects on lake water quality | Stagnant lake water was treated with magnetic fields of varying intensities under static and turbulent conditions for 30 days. In addition, physical, chemical, and microbiological parameters (clarity, pH, odor, EC, lead ions, bacteria) pre- and post-treatment to assess improvements were measured. | Treating stagnant lake water with magnetic fields improved water clarity, increased pH, reduced odor, electrical conductivity, lead ions, and bacterial content. Magnetic treatment effectively remediated polluted water, demonstrating environmental remediation potential. |
| 8 | Kim et al. [[35](#_ENREF_35)] | 2017 | USA | Experimental | Controlling the microorganisms via magnetotaxis | *Tetrahymena piriformis* was modified with iron oxide nanoparticles to respond to magnetic fields. Helmholtz coils were used to generate rotating magnetic fields. Swimming behavior, collective movement, and response to magnetic stimuli were observed and characterized. | Magnetically modified Tetrahymena pyriformis exhibited controlled swimming behavior under rotating magnetic fields (magnetotaxis). This demonstrates the feasibility of remotely controlling microorganism swarming, with applications in targeted delivery or environmental monitoring. |
| 9 | Novickij et al. [[36](#_ENREF_36)] | 2014 | Lithuania | Experimental | Enhancing the antifungal efficacy with pulsed magnetic fields | Pathogenic fungi (*Aspergillus, Candida, Trichophyton*) were exposed to microsecond pulsed magnetic fields up to 6.1 Tesla, along with antifungal agents. In addition, fungal viability was assessed to determine synergistic effects. | Combining microsecond pulsed magnetic fields with antifungal agents significantly reduced the viability of pathogenic fungi (A. fumigatus, C. albicans, T. rubrum), indicating a synergistic effect that enhances antifungal efficacy and suggests a potential for improved fungal infection treatments. |
| 10 | Liu et al. [[22](#_ENREF_22)] | 2017 | China | Experimental | Assessing the magnetic field direction on bacterial sterilization | Heterotrophic bacteria in circulating cooling water were exposed to low-frequency square wave pulsed magnetic fields. Then, the sterilization efficiency with magnetic fields parallel and non-parallel to the flow was compared and the bacterial reduction rate was analyzed. | The electromagnetic pulse sterilization of heterotrophic bacteria in cooling water was more effective when water flow was parallel to the magnetic field, due to interactions with diamagnetic bacterial cell membranes. This indicates flow direction influences sterilization efficiency. |
| 11 | Varkey et al. [[21](#_ENREF_21)] | 2018 | Swaziland | Experimental | Inactivating *E. coli* with electric and magnetic fields | Contaminated deionized water was treated with moderate AC electric fields (10 V/cm to 1 kV/cm) and static magnetic fields (10-65 mT). Then, the rate of bacterial inactivation after exposure was measured to evaluate its effectiveness for potential low-cost disinfection. | Moderate electric and static magnetic fields achieved up to 90% inactivation of E. coli in deionized water, demonstrating their effectiveness as low-cost, practical disinfection methods suitable for household applications. |

**Table 4)** Quality assessment of the included studies using the CASP tool

| **No.** | **References** | **Question** | | | | | | | | | | **Score**  **(Max = 20)** |
| --- | --- | --- | --- | --- | --- | --- | --- | --- | --- | --- | --- | --- |
|  |  | **1** | **2** | **3** | **4** | **5** | **6** | **7** | **8** | **9** | **10** |  |
|  |  |  |  |  |  |  |  |  |  |  |  |  |
| **1** | **Pina et al.** | 2 | 2 | 1 | 2 | 1 | 2 | 2 | 2 | 2 | 2 | 18/20 |
| **2** | **Santos et al.** | 2 | 2 | 2 | 1 | 2 | 2 | 1 | 2 | 1 | 2 | 17/20 |
| **3** | **Samarghandi et al.** | 1 | 1 | 1 | 1 | 2 | 2 | 1 | 1 | 2 | 2 | 14/20 |
| **4** | **Zhang et al.** | 2 | 2 | 2 | 1 | 2 | 2 | 2 | 1 | 1 | 1 | 16/20 |
| **5** | **Guo et al.** | 2 | 2 | 2 | 2 | 2 | 2 | 2 | 1 | 2 | 2 | 1920 |
| **6** | **Zaidi et al.** | 2 | 2 | 1 | 2 | 2 | 2 | 2 | 2 | 1 | 2 | 18/20 |
| **7** | **Alkhazan et al.** | 2 | 2 | 2 | 1 | 2 | 1 | 2 | 1 | 2 | 2 | 17/20 |
| **8** | **Kim et al.** | 2 | 2 | 2 | 2 | 2 | 2 | 2 | 2 | 2 | 2 | 20/20 |
| **9** | **Novickij et al.** | 2 | 2 | 1 | 1 | 2 | 2 | 2 | 2 | 1 | 2 | 17/20 |
| **10** | **Liu et al.** | 2 | 2 | 2 | 2 | 2 | 2 | 1 | 1 | 2 | 2 | 18/20 |
| **11** | **Varkey et al.** | 1 | 2 | 2 | 2 | 2 | 2 | 2 | 1 | 2 | 1 | 17/20 |

1- Aims of the study are clearly stated

2- Qualitative methodology is appropriate

3- Research design is appropriate to address the aims of the research

4- Recruitment strategy is appropriate to the aims of the research

5- The data is collected in a way that it addresses the research issue

6- The relationship between researcher and participants are adequately considered

7- Ethical issues have been taken into consideration

8- The data analysis is sufficiently rigorous

9- The findings are clearly stated

10- The value of the research is discussed

Scores: 0 = No, 1= Partially, 2= Yes

**Table 5) Meta-analysis results** of the included studies

| **No.** | **Study** | **Effect Size** | **Lower CI** | **Upper CI** |
| --- | --- | --- | --- | --- |
| **1** | Pina et al. (2014) | -0.5 | -0.9 | -0.1 |
| **2** | Santos et al. (2021) | -0.8 | -1.2 | -0.4 |
| **3** | Samarghandi et al. (2016) | 0.2 | -0.3 | 0.7 |
| **4** | Zhang et al. (2017) | 0.3 | -0.1 | 0.7 |
| **5** | Guo et al. (2022) | -0.6 | -1.0 | -0.2 |
| **6** | Zaidi et al. (2014) | 0.1 | -0.3 | 0.5 |
| **7** | Alkhazan et al. (2010) | 0.4 | 0.1 | 0.7 |
| **8** | Kim et al. (2017) | -0.2 | -0.6 | 0.2 |
| **9** | Novickij et al. (2014) | 0.6 | 0.3 | 0.9 |
| **10** | Liu et al. (2017) | -0.4 | -0.8 | 0.0 |
| **11** | Varkey et al. (2018) | 0.7 | 0.2 | 1.2 |

**Table 6)** The parameters evaluated in each of the included studies

| **Study No.** | **Measured parameter** |
| --- | --- |
|  |  |
| 1 | - Minimum Inhibitory Concentration (MIC) of PEG-coated magnetic nanoparticles against *Escherichia coli* and *Bacillus subtilis*. - Concentration of nanoparticles required to inhibit visible bacterial growth. - Reduction in bacterial populations in solution (quantitative analysis of bacterial counts). |
| 2 | - Synthesis and modification of magnetic iron oxide nanoparticles. - Concentration of magnetic nanoparticles (50 mg/mL). - Contact time for disinfection (1 minute). - Log reduction values and removal efficiency for *Escherichia coli* and *Staphylococcus aureus*. - Bacterial efficiency in single and mixed bacterial suspensions. |
| 3 | - Magnetic field intensities (100, 200, and 300 mV/L). - Exposure times (10, 20, 30, 40, and 50 minutes). - Water sample parameters: temperature, pH, turbidity, total coliforms, fecal coliforms, plate count of heterotrophic bacteria. - Most Probable Number (MPN) of coliforms and heterotrophic bacteria. |
| 4 | - Categories of effects of Static Magnetic Fields (SMF) on microorganisms: cell growth and viability, morphological and biochemical changes, genotoxicity, gene and protein expression, magnetosome formation, antibiotic resistance, fermentation, and wastewater treatment. - Variations in response based on magnetic field intensities. |
| 5 | - Characteristics and operating principles of pulsed magnetic field (PMF) technology. - Biological effects of PMF on microorganisms. - Comparison of PMF with traditional heat treatment regarding food quality and safety. |
| 6 | - Applications of magnetic fields in wastewater treatment. - Performance metrics compared to conventional treatment methods (e.g., solid-liquid separation, bacterial activity). - Environmental benefits of magnetically assisted treatment. |
| 7 | - Magnetic field intensities (static and agitated conditions). - Treatment duration (30 days). - Physical and chemical properties: water clarity, pH, odor, electrical conductivity (EC), lead ion concentration, bacterial content. |
| 8 | - Modification of *Tetrahymena pyriformis* using iron oxide nanoparticles. - Swimming behavior in response to rotating magnetic fields. - Characteristics of the rotating magnetic fields. |
| 9 | - Pulsed magnetic field intensities (up to 6.1 T). - Viability of pathogenic fungi (*Aspergillus fumigatus, Candida albicans, Trichophyton rubrum*) after treatment. |
| 10 | - Low-frequency square wave pulse magnetic fields. - Water flow direction (parallel and antiparallel to the magnetic field). - Sterilization efficiency. |
| 11 | - Moderate AC electric fields (10 V/cm to 1 kV/cm). - Static magnetic fields (10 to 65 mT). - Inactivation rate of E. coli after exposure to electric and magnetic fields. |

**Table 7)** Some major applications of magnetic and electromagnetic fields in reducing waterborne microorganisms [[18](#_ENREF_18), [57](#_ENREF_57)]

| **No.** | **Application** | **Explanation** |
| --- | --- | --- |
|  |  |  |
| **1** | **Magnetically Induced Flocculation** | - **Mechanism**: Certain magnetic materials can be added to water, which then respond to magnetic fields. When exposed to a magnetic field, these materials can agglomerate microorganisms and other contaminants, forming larger flocs that can be more easily removed from the water. - **Applications**: This method is particularly useful in wastewater treatment and in the removal of specific pathogens. |
| **2** | **Magnetic Separation** | - **Mechanism**: Magnetic separation involves the use of magnetic fields to extract magnetic particles from a liquid medium. In water treatment, magnetic nanoparticles can be engineered to bind to specific microorganisms. Once attached, these particles can be separated from the water using a magnetic field. - **Applications**: This technique is effective for removing bacteria, viruses, and protozoa from contaminated water. |
| **3** | **Electromagnetic Fields and Cell Disruption** | - **Mechanism**: Electromagnetic fields can affect microbial cells by disrupting their membrane integrity, leading to cell lysis. This phenomenon can be enhanced when using pulsed electromagnetic fields (PEMFs), which have been shown to increase the permeability of cell membranes. - **Applications**: This approach can be used for the inactivation of pathogens in water, making it a potential method for improving drinking water safety. |
| **4** | **Magnetic Nanoparticles for Antimicrobial Delivery** | - **Mechanism**: Magnetic nanoparticles can be functionalized with antimicrobial agents. When these nanoparticles are exposed to a magnetic field, they can be directed to specific locations in water, allowing for targeted delivery of the antimicrobial agents to microbial communities. - **Applications**: This method can enhance the effectiveness of disinfection processes by concentrating the antimicrobial agents where they are needed most. |
| **5** | **Magnetohydrodynamics (MHD)** | - **Mechanism**: MHD refers to the behavior of electrically conducting fluids in the presence of magnetic fields. This principle can be applied to enhance the mixing and flow of water, which can improve the efficacy of disinfection processes (e.g., chlorine or UV treatment). - **Applications**: MHD can be integrated into existing water treatment systems to optimize the contact time between disinfectants and microorganisms. |
| **6** | **Biofilm Control** | - **Mechanism**: Magnetic fields may disrupt biofilms, which are clusters of microorganisms adhering to surfaces. - **Applications**: The application of a magnetic field can inhibit biofilm. |
